# Potential Application of MicroRNA Profiling to the Diagnosis and Prognosis of HIV-1 Infection

**DOI:** 10.3389/fmicb.2018.03185

**Published:** 2018-12-21

**Authors:** Bin Su, Yuping Fu, Yan Liu, Haoquan Wu, Ping Ma, Weiping Zeng, Tong Zhang, Shi Lian, Hao Wu

**Affiliations:** ^1^Center for Infectious Diseases, Beijing Youan Hospital, Capital Medical University, Beijing, China; ^2^Beijing Key Laboratory for HIV/AIDS Research, Beijing, China; ^3^Department of Dermatology, Xuanwu Hospital, Capital Medical University, Beijing, China; ^4^Kanglin Biotech (Hangzhou) Co., Ltd., Zhejiang, China; ^5^Department of Infectious Diseases and STDs, Tianjin Second People’s Hospital, Tianjin, China; ^6^Department of Biochemistry and Microbiology, Marshall University School of Medicine, Huntington, WV, United States

**Keywords:** HIV-1, microRNA, disease progression, biomarker, immune system

## Abstract

MicroRNAs (miRNAs) were first identified in *Caenorhabditis briggsae* and later recognized as playing pivotal roles in a vast range of cellular activities. It has been shown that miRNAs are an important mechanism not only for host defense against virus but also for the establishment of viral infection. During human immunodeficiency virus type 1 (HIV-1) infection, host miRNA profiles are altered either as a host response against the virus or alternatively as a mechanism for the virus to facilitate viral replication and infection or to maintain latency. The altered miRNA profiles can be detected and quantified by various advanced assays, and potentially serve as more sensitive, accurate and cost-efficient biomarkers for HIV-1 diagnosis and disease progression than those detected by currently available standard clinical assays. Such new biomarkers are critical for optimizing treatment regimens. In this review, we focus on the potential application of miRNA profiling to the diagnosis of HIV-1 infection and the monitoring of disease progression.

## Introduction

**FIGURE 1:**
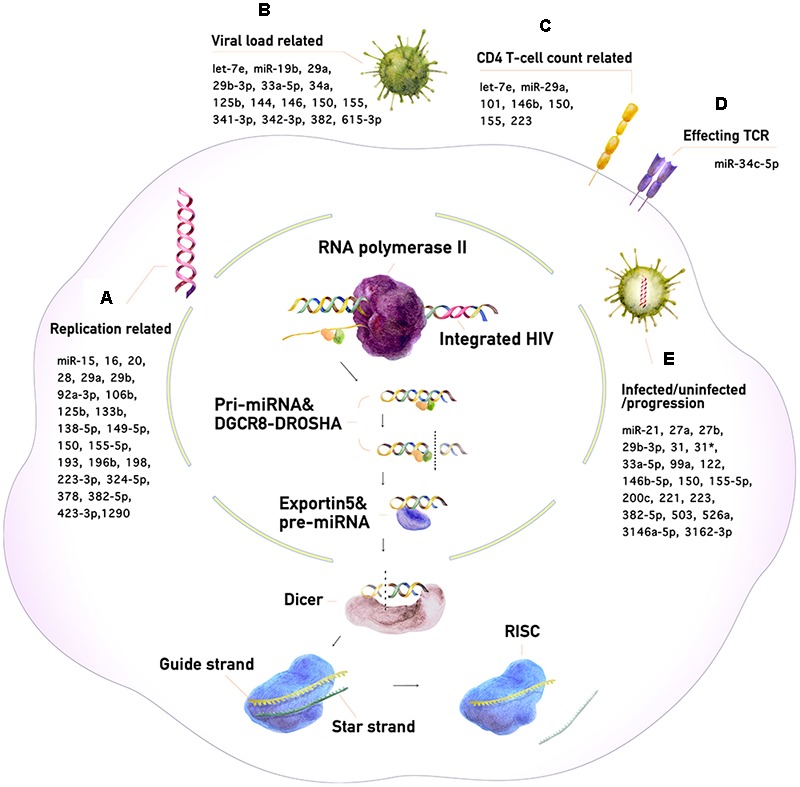
The miRNA biogenesis pathway and its impact on HIV-1 infection. **(A)** Enhancement or suppression of HIV-1 replication by miRNAs. **(B)** miRNAs correlating with HIV-1 viral load. **(C)** miRNAs correlating with CD4^+^ T-cell counts. **(D)** miRNAs affecting the T-cell receptor (TCR). **(E)** miRNAs for the diagnosis of HIV-1 infection and monitoring of its progression.

MicroRNAs (miRNAs) are small non-coding RNAs that regulate gene expression. In the human genome, more than 1500 different miRNAs have been identified, which collectively regulate the expression of about one third of all human genes (Li and Zhang, 2013). miRNAs regulate diverse biological functions, such as cellular differentiation, development, apoptosis, tumorigenesis, and targeting of foreign pathogens (Ouellet et al., 2009; Berkhout and Liu, 2014). miRNA research has advanced considerably in recent years. It has been shown that the expression of many miRNAs is altered during human immunodeficiency virus type 1 (HIV-1) infection. Changes in miRNA expression in infected humans can be detected with various advanced assays (Affymetrix^TM^ assay, digital droplet qPCR, *etc*., and sequencing). Therefore, miRNAs can potentially be used to detect HIV-1 infection and monitor disease progression with greater sensitivity and efficiency than conventional methods. This review provides an overview of current knowledge concerning miRNA and its relation to HIV-1 infection and disease progression. A model of the miRNA biogenesis pathway and its correlation with CD4^+^ T-cell count, T-cell receptor expression, serum viral load, and virus replication is proposed in Figure 1.

## The miRNA Biogenesis Pathway

The genes encoding miRNAs are dispersed throughout the human genome (Rodriguez et al., 2004). Many miRNAs are generated as the sole transcripts of non-coding regions. Genes encoding miRNAs may also occur within introns or untranslated regions of protein-coding DNA (Poliseno et al., 2010; Ramalingam et al., 2014). The genes transcribed by RNA polymerase II can produce messenger RNAs (mRNAs) or regulatory RNAs called primary RNAs (Lee et al., 2004), which form a typical hairpin loop structure and undergo several processing steps to become miRNAs (Quick-Cleveland et al., 2014). The processing begins with recognition of the double-stranded stem by the DiGeorge syndrome chromosomal region 8 (DGCR8) protein. An enzyme called DROSHA then associates with DGCR8 to form a complex that cleaves the RNA to generate a smaller precursor miRNA (Yang and Lai, 2011; Wu et al., 2012). The precursor miRNA is transported from the nucleus to the cytoplasm by the transporter protein exportin 5 and is then immediately recognized by an RNase called Dicer. Dicer cleaves the hairpin loop structure to form a short double-strand miRNA molecule. The Argonaut protein then interacts with Dicer and binds the miRNA. The miRNA is unwound and one strand, the “star strand” or “passenger strand,” is released and degraded. The remaining strand, the “guide strand,” which is typically 19–22 nucleotides long, interacts with several proteins to form the RNA-induced silencing complex (RISC). The guide strand guides the RISC complex to its target sequences in mRNA, enabling it to inactivate one or several genes. The mRNA of the target gene is complementary to the essential “seed” region of the miRNA, which is often 2∼8 nucleotides long, facilitating partial complementary base pairing with the mRNA. Once bound, the RISC can block mRNA translation by preventing the ribosome subunit from binding to the mRNA. As a result, the mRNA is not translated into protein and the gene is effectively silenced (Swaminathan et al., 2012a; Fukaya et al., 2014; Zinovyeva et al., 2015). The vital role of miRNA in a vast range of biological processes opens up tremendous possibilities for new treatments and diagnosis of various diseases in humans. We focus here on the potential of miRNA for the diagnosis of HIV-1 infection and monitoring of its progression.

## The Role of miRNAs in HIV-1 Infection

HIV-1 mainly infects human CD4^+^ T cells through the recognition of the CD4^+^ receptor on the cell surface. The virus gradually reduces the number of CD4^+^ T cells in the infected patient, which impairs the human immune system and eventually leads to acquired immunodeficiency syndrome (AIDS) (Weiss, 1993; Douek et al., 2009). After gaining entry to the body *via* the intravenous route or lesions in mucosal surfaces, HIV-1 binds to the CD4 receptor and the CCR5 or CXCR4 co-receptors on immune cells. The virus then penetrates the cell membrane and releases its RNA genome into the cell, which is subsequently reverse-transcribed into DNA and integrated into the host cell genome (Haase, 2011). Viral particles are produced in the infected cells, and disseminate from the initial site of infection to establish systemic infection. At late stages of infection, which are characterized by high viral load and low CD4^+^ T-cell counts in the blood, the patient becomes susceptible to infection with secondary pathogens, such as *Pneumocystis carinii, Candida albicans*, and *Mycobacterium tuberculosis*, eventually succumbing to AIDS (Aaron et al., 2004). HIV-1 replication within host cells is regulated by various host factors, including miRNAs, which may target virus mRNAs directly or regulate the expression of host proteins that HIV-1 hijacks for its own replication. The miRNA profile of the host changes during infection, with different miRNAs produced at different stages of disease progression in HIV-1-infected individuals (Swaminathan et al., 2012a).

## The Effect of HIV Infection on miRNA Expression

HIV-1 infection is known to trigger changes in the expression of many miRNAs, either by influencing host cellular activities or as a result of the cellular response to HIV-1 infection (Balasubramaniam et al., 2018). The ability of miRNA analyses to distinguish between individuals with and without HIV-1 infection has been reported worldwide. In 2012, Gupta et al. (2011) analyzed 704 kinds of miRNA in peripheral blood mononuclear cells (PBMCs) of HIV-1-infected individuals and healthy controls. They found that 28 of these miRNAs were up-regulated and 14 were down-regulated in HIV-infected individuals. Using Target Scan software, they predicted that the targets of these miRNAs were mostly related to the cell cycle, proliferation, movement, migration, and signal transduction. The altered transcriptional profiles of these miRNAs may lead to various non-AIDS disorders (Gupta et al., 2011). On the other hand, HIV-1 gp120 and tat were recently shown to accelerate endothelial cell senescence by disrupting senescence-associated miRNAs, thereby increasing the risk of atherosclerotic vascular disease. This discovery provides further support for the notion that, as CD4^+^ T cells are the main targets of HIV-1 infection, they should contain miRNAs that are differentially expressed in the presence and absence of HIV-1 infection. Swaminathan’s team demonstrated significant down-regulation of the Let-7 family of miRNAs in patients with chronic HIV-1 infection (Swaminathan et al., 2012b). In the same year, a study on elite controllers led by Witwer et al. (2012) showed that miR-125b and -150 levels were strongly correlated with HIV-1 viral load in HIV-1-infected patients. Amaral et al. (2017) recently discovered that levels of miR-34c-5p, a miRNA responding to T-cell receptor stimulation in naïve T cells, were decreased during the disruption of host immune responses by HIV-1. In 2014, researchers analyzed subcutaneous adipose tissue from eight HIV-1-infected patients. They used human miRNA microarrays to probe the transcriptomes of the samples and found that 21 of 754 miRNAs were overexpressed, with 10 of these miRNAs displaying at least 2.5-fold up-regulation (Squillace et al., 2014). Thapa et al. (2014) suggested that serum miR-21, miR-122, and miR-223 levels can be used to distinguish between HIV-1-infected and uninfected individuals, and that miR-222 can also be used as a diagnostic marker for AIDS-associated non-Hodgkin lymphoma.

## The Role of miRNAs in HIV-1 Replication

MicroRNAs can affect HIV-1 infection through multiple mechanisms. Anti-HIV-1 miRNAs repress HIV-1 activities through CCR5 or CXCR4, the auxiliary receptors for HIV-1, or target HIV-1 directly *via* the *env*, *pol*, *gag*, *vif*, and *tat* genes of the HIV-1 genome. Hariharan et al. (2005) used a consensus scoring method and showed that miR-29a and miR-29b targeted *nef*, miR-149 targeted *vpr*, miR-378 targeted *env*, and miR-324-5p targeted *vif* within the HIV-1 genome. Huang et al. (2007) discovered that miR-28, miR-125b, miR-150, miR-223, and miR-382-5p targeted the 3′UTR region of the HIV-1 mRNA in cells, which decreases CD4^+^ T-cell activation during the resting period (Huang et al., 2007). Monocytes are rich in these anti-HIV-1 miRNAs, which are down-regulated in macrophages after differentiation, rendering macrophages susceptible to HIV-1 infection. The suppression of these miRNAs increases the susceptibility of mononuclear cells to HIV-1 infection (Hariharan et al., 2005; Huang et al., 2007). The 3′UTR region of the HIV-1 RNA genome has been identified as the target of miR-196b and miR-1290 (Wang et al., 2015). The inhibition of these two miRNAs can lead to the activation of latent HIV-1, leading to viral clearance of latent viral reservoirs by virus-induced cytolysis and host antiviral immune responses in the presence of antiretroviral therapy (ART) (Mbonye and Karn, 2014; Wang et al., 2015, 2018).

Some miRNAs can also enhance HIV-1 infection. Chiang et al. (2013) found that miR-132 was significantly more strongly expressed in activated CD4^+^ T cells than in steady-state CD4^+^ T cells. Although the underlying mechanism for this remains unknown, in this study they found that miR-132 enhanced HIV-1 replication and increased the susceptibility of activated CD4^+^ T cells to HIV-1 infection (Chiang et al., 2013). miR-217 and miR-34a can significantly increase tat levels, and can also bind to mRNA of the *SIRT1* gene (Kwon and Ott, 2008) and inhibit its expression, thereby enhancing the transcriptional activation mediated by HIV-1 tat (Zhang et al., 2012).

MicroRNAs also indirectly regulate the effects of HIV-1 infection by targeting the HIV-dependent factors interacting with HIV-1. The histone acetyltransferase P300 and P300-CREB binding protein-associated factor (PCAF) are required for tat acetylation, leading to the up-regulation of the transcription of the HIV-1 LTR. Cell-associated miR-17/92 targets PCAF and inhibits viral replication (Triboulet et al., 2007). miR-198 plays an anti-HIV-1 role by down regulating cyclin T1, an important component of the eukaryotic RNA polymerase II prolongation complex. This protein forms a heterodimer with CDK9 (transcription elongation factor B or p-TEFb). The p-TEFb complexes interact with HIV-1 transactivating response (TAR) elements and tat, promoting viral transcription. Cyclin T1 is more strongly expressed in HIV-1-susceptible cells, such as macrophages, than in mononuclear cells (Sung and Rice, 2009). Pur-alpha is a sequence-specific DNA and RNA binding protein that binds to the HIV-1 TAR element and tat in HIV-1-infected nuclei, up regulating viral transcription (Chepenik et al., 1998). Unlike macrophages and dendritic cells, which differentiate from monocytes, monocytes themselves displayed a significant repression of Pur-alpha. This repression is due to the inhibitory effect of certain miRNAs such as miR-15a, miR-15b, miR-16, miR-20a, miR-193, and miR-106b targeting Pur-alpha, and consequentially decreased the susceptibility to HIV-1 infection of monocytes compared to their macrophage “descendants” (Shen et al., 2012).

More interestingly, some studies have reported that the interference capacity of miRNAs is not restricted to gene silencing. Some miRNAs can bind to the nucleocapsid protein. Research led by Chen discovered that certain miRNAs interfere with HIV-1 replication by binding to the gag protein and intercepting HIV-1 assembly, rather than by forming RISCs and recognizing mRNAs (Chen et al., 2014).

Most of the mentioned miRNAs function by suppressing HIV-1 replication, but others may promote this process. Either way, the shift in miRNA profile caused by HIV-1 infection or as part of the host immune response indicates a change in the capability of HIV-1 to replicate that is closely related to the viral load.

## HIV-1-Encoded miRNAs

Viral genomes may also encode their own miRNAs, which could be even more useful than human miRNAs for the diagnosis of viral infection. The first viral miRNAs to be described, in 2004, were from Epstein-Barr virus (Pfeffer et al., 2004). However, given that HIV-1 is an RNA virus, it remains unclear whether it encodes its own miRNAs. A well accepted view is that the RNA-based HIV-1 genome might be inactivated by its own miRNAs or the intermediate products during the processing of these miRNAs, thereby interfering with the replication of these miRNA-encoding sequences (Umbach et al., 2010; Whisnant et al., 2013; Harwig et al., 2014). Nevertheless, evidence for HIV-1-encoded miRNAs has been accumulating over the last few years. These viral miRNAs may play a role in the versatility of the viruses and their ability to hijack host cellular pathways for their own purposes.

The first candidates of HIV-1-encoded miRNAs were identified in an *in silico* analysis by Bennasser et al. (2004) only a few months after the description of the first viral miRNA. This analysis of the HIV-1 genome predicted a few possible viral miRNAs encoded by HIV-1 (Bennasser et al., 2004). Couturier and Root-Bernstein (2005) subsequently suggested that HIV may produce miRNAs that inhibit the expression of some cell surface markers of clusters of differentiation (CD) and interleukins (IL). More recently, HIV-1 has been shown to produce a replication-enhancing miRNA from its reverse transcriptase region (Zhang et al., 2014). The debate about whether or not HIV-1 can produce its own miRNA thus continues, with evidence on both sides, but these potential viral miRNAs merit further investigation.

## Changes in miRNA Levels in PBMCs and Serum From Patients Based on HIV Disease Progression

In the studies led by Houzet, the patients were assigned to four groups on the basis of CD4^+^ T-cell counts, viral load, and the miRNA expression profiles of PBMCs in HIV-1-infected individuals. In total, 63 different miRNAs displayed changes in levels in the HIV-1-infected PBMCs, indicating differential expression of specific miRNAs among the different groups. For example, let-7e, miR-101, miR-146b, and miR-155 were found only in the low CD4^+^ T-cell count and high-viral load group. The levels of miR-223, miR-150, miR-146b, miR-16, and miR-191 in T cells were lower, by a factor of three to nine, in the PBMCs of patients with different disease progression profiles. This observation suggested that specific miRNAs are potential biomarkers for differentiating the stages of progression of HIV-1 infection (Houzet et al., 2008). Dey et al. (2016) compared the expression of miRNAs in PBMCs between long-term non-progressors (LTNP), slow progressors, and rapid progressors. Levels of miR-382-5p were lower in LTNP and displayed a positive linear correlation with plasma viral load. A combination of miR-382-5p and miR-155-5p can be used to distinguish between LTNP and chronic progressors, and between infected and non-infected individuals. Decreases in the levels of miR-382-5p and miR-155-5p are beneficial for the control of disease progression (Dey et al., 2016).

About 10∼15% of HIV-1-infected patients display a rapid decrease in CD4^+^ T-cell count at early stages of infection, with rapid progression to AIDS. A comparison of the miRNA expression profiles in the PBMCs of rapid progressors and slow progressors revealed differences in miR-31, miR-200c, miR-526a, miR-99a, and miR-503 levels that could be used as biomarkers for rapid progressors, favoring early intervention (Sung and Rice, 2009). Duskova et al. (2013) found that miR-19b, miR-146a, miR-615-3p, miR-382, miR-34a, miR-144, and miR-155, which regulate innate immunity- and inflammation-related genes, were significantly up-regulated in PBMCs with a high viral load in HIV-1-infected patients.

Munshi et al. (2014) compared the levels of expression of miR-16, miR-146b-5p, miR-150, miR-191, miR-223, and miR-146b-5p in plasma between patients without AIDS, patients with AIDS who had not received ART, patients who had received ART and patients who had received ART but displayed drug resistance. They found that miR-146b-5p levels in PBMCs and plasma not only distinguished different progression groups, but also predicted reactivity to ART. In addition, miR-150 could be used as an index for monitoring the progression of HIV/AIDS disease and the effect of ART (Munshi et al., 2014). Rosca et al. (2016) found that levels of miR-29a were inversely correlated with HIV viral load and the degree of immunosuppression (the number of CD4^+^ T cells and CD4^+^ T/CD8^+^ T ratio) in 165 young subjects with chronic HIV-1 infection. Levels of miR-29a and responses to treatment are different among patients. Levels of miR-29a were low in patients who had experienced treatment failure (CD4 < 350 cells/μl). miR-29a thus can be used as a biological marker of long-term survivors and patients who have received ART, and it can also be used to predict disease prognosis and progression (Rosca et al., 2016).

The small proportion of elite controllers among HIV-infected patients can effectively control the infection without drug treatment. Elite controllers are infected with HIV but able to sustain undetectable viral loads and usually normal CD4^+^ T-cell count without any treatment (Walker, 2007; Autran et al., 2011). Witwer et al. (2012) found that a difference in miR-31 and miR-31^∗^ levels between the PBMCs of patients with HIV-1 viremia and elite controllers reflected the progression of HIV-1 infection. miR-31 levels were positively correlated with CD4^+^ T-cell count, whereas miR-341-3p levels were negatively correlated with viral load. This result suggests the levels of these miRNAs are potential biomarkers of the risk of progression of HIV-1 infection (Witwer et al., 2012). Reynoso et al. (2014) compared the changes in plasma levels of miRNAs in elite controllers, individuals with chronic HIV-1 infection and healthy individuals. They found that plasma miR-29b-3p, miR-33a-5p, and miR-3146a-5p levels in elite controllers were significantly higher than those in patients with chronic infection. Further experiments *in vitro* showed that the overexpression of miR-29b-3p and miR-33a-5p could reduce HIV-1 production. Egana-Gorrono et al. (2014) analyzed the expression profiles of 286 different human miRNAs in elite controllers, individuals with viremia, individuals who received ART and healthy individuals: 23 different miRNAs displayed differential expression between the elite controller and viremia groups. These miRNAs included miR-221, miR-27a, miR-27b, and miR-29b, which were up-regulated in the serum of elite controllers and HIV-1-negative individuals, but were down-regulated in the serum of patients with viremia and HIV-1-infected patients on ART. Moreover, another 19 different miRNAs were down-regulated in the serum of elite controllers and HIV-1-negative individuals, and up-regulated in the serum of individuals with viremia and HIV-1-infected individuals on ART (Egana-Gorrono et al., 2014).

The change of miRNA profiles in CD4^+^ T cells is even more closely correlated with disease progression since the CD4^+^ T cells are the main targets of HIV-1 infection. Interferon-inducible protein 10 (IP-10) is a crucial inflammatory cytokine that can trigger immune dysfunction and disease progression during HIV-1 infection. By comparing the sequences of the gene encoding IP-10 and those of human miRNAs, Wu et al. (2017) recently found that six miRNAs matched the sequence of the IP-10 3′UTR. Further analysis revealed that one of these miRNAs, miR-21, was down-regulated in monocytes during HIV-1 infection and responsible for the increase in IP-10 levels leading to inflammation and the rapid loss of CD4^+^ T cells, which is closely related to disease progression (Wu et al., 2017).

## The Relationship Between miRNAs and Disease Progression in HIV-1-Infected Individuals

MicroRNA has been shown to correlate with viral latency and disease progression. Many researchers found distinct miRNA profile during different stages of disease progression, and levels of certain miRNAs may influence the susceptibilities of certain cells (as discussed in the section regarding PBMCs and serum) as well as the speed of disease progression period.

Host miRNA is very important for HIV-1 latency. For example, the CD4^+^ T cells of HIV-1-infected individuals in the LTNPs and untreated patients groups display changes in miR-155 level (Bignami et al., 2012). The Let-7 family of miRNAs targets IL-10, a pleiotropic cytokine. Let-7 is down-regulated in chronic progressors (Zhang et al., 2016). IL-2 is also a major factor for the maintenance of the activation, survival and replication of CD4^+^ T cells. The levels of miR-9 in CD4^+^ T cells from individuals with chronic HIV-1 infection are lower than those in healthy individuals or LTNPs. This decrease in miR-9 levels triggers an increase in mature protein 1 in B cells, leading to the inhibition of IL-2 production and promoting disease progression, which is also evidence for the positive role of miR-9 in maintaining HIV-1 latency (Seddiki et al., 2013). These findings suggest that long-term monitoring of miR-155 might be very valuable for assessing HIV-1 latency status in infected individuals.

Many other factors are correlated with immune status and disease progression. The abundance and size of extracellular vesicles are correlated with the apoptosis and pyroptosis of CD4^+^ T cells and with miR-155 and miR-223 levels, as shown by Hubert et al. (2015). As the authors suggested, analysis of these miRNAs, together with plasma exosome abundance, extracellular vesicles and size may provide accurate fingerprint of disease progression (Hubert et al., 2015).

The expression of miRNAs in cells and body fluids has been shown to be associated with the severity and progression of HIV infection. On the basis of CD4^+^ T-cell counts, viral load and whether ART has been administered, HIV-1-infected patients can be divided into typical progressors, rapid progressors, slow progressors, LTNP and HIV-infected individuals in the early stages of antiretroviral treatment (Li et al., 2010). Some miRNAs are differentially expressed in these different groups, and theirabnormal expression can serve as a biomarker of the disease progression and provide important information about prognosis (Dey et al., 2016).

For instance, Munshi’s team described miR-150 as a potential biomarker for HIV/AIDS disease progression and treatment monitoring. They compared its levels in PBMCs and plasma between healthy donors, asymptomatic patients, symptomatic patients, patients on ART and patients who had developed drug resistance. They found that this particular miRNA was differentially expressed among these groups (Munshi et al., 2014). Similarly, in a very recent study, Huang et al. (2018) collected plasma from healthy donors, patients infected for less than 1 year and patients infected for more than 1 year. Differential expression of miR-3162-3p was observed between these groups. The clinical significance of this discovery was validated by successfully predicting disease progression based on the expression of the miRNA and in an *in vitro* model (Huang et al., 2018).

## Concluding Remarks and Remaining Questions

This review highlights the value of miRNA for the diagnosis of HIV-1 infection and the evaluation of disease progression. We have considered the effects of miRNA on HIV-1 replication and of HIV-1 infection on miRNA profiles in different cell subsets. In each case, there is growing evidence for differences in miRNA levels directly or indirectly due to HIV-1 infection. While standard testing methods have their limitations such as in detecting low viral load, monitoring miRNA may provide valuable information for diagnosis and predicting HIV/AIDS progression. It may now be feasible to establish a national or international database or guideline for miRNA-based disease diagnosis. However, there are still many obstacles. Some of the studies mentioned in this article suggest that miRNA profiles are not entirely homogeneous within a given group and that many miRNA levels are individual-dependent, with changes in their profiles not directly due to HIV-1 infection. In addition, some of the studies described here were performed *ex vivo* and confirmation of their results is required *in vivo* and in clinical trials. Furthermore, according to some reports, HIV-1 may also be able to develop resistance to miRNA interference (Boden et al., 2003; Westerhout et al., 2005), which would complicate the pursuit of approaches based on the use of miRNA biomarkers. With the development of highly sensitive assays and high-throughput sequencing, advances are being made in the field of miRNAs. While there are still some pieces missing from the puzzle before miRNAs can be used as clinical biomarkers for HIV-1 infection and HIV/AIDS disease progression, many studies, as reviewed in this article, have demonstrated the potentials of miRNAs as biomarkers for these clinical processes.

## Author Contributions

All authors listed have made a substantial, direct, and intellectual contribution to the work and read and approved the final manuscript.

## Conflict of Interest Statement

The authors declare that the research was conducted in the absence of any commercial or financial relationships that could be construed as a potential conflict of interest.
